# Supplementary material for: Phytochemical Investigation of Tradescantia Albiflora and Anti-Inflammatory Butenolide Derivatives
Source: Molecules. 2019 Sep 13;24(18):3336. doi: 10.3390/molecules24183336 (PMC6767271; doi:10.3390/molecules24183336)
Supplement: Supplementary file 1 [file molecules-24-03336-s001.pdf]

Supplementary Materials

Phytochemical investigation of *Tradescantia albiflora* and anti-inflammatory butenolide derivatives

Ping-Chen Tu [1], Han-Chun Tseng [2], Yu-Chia Liang [3], Guan-Jhong Huang [3], Te-Ling Lu [4,†], Tzong-Fu Kuo [5,†] and Yueh-Hsiung Kuo [3,6,7,*]

[1] The Ph.D. Program for Cancer Biology and Drug Discovery, China Medical University and Academia Sinica, Taichung 404, Taiwan
[2] Department of Chemistry, National Taiwan University, Taipei 106, Taiwan
[3] Department of Chinese Pharmaceutical Sciences and Chinese Medicine Resources, China Medical University, Taichung 404, Taiwan
[4] School of Pharmacy, China Medical University, Taichung 404, Taiwan
[5] Department of Post-Baccalaureate Veterinary Medicine, Asia University, Taichung 413, Taiwan
[6] Department of Biotechnology, Asia University, Taichung 413, Taiwan
[7] Chinese Medicine Research Center, China Medical University, Taichung 404, Taiwan
[†] The authors contributed equally to this work
[*] Correspondence: kuoyh@mail.cmu.edu.tw; Tel.: +886-4-2205-3366ext.5709 (Y.-H.K.)

Figure S1. IR spectrum of compound **1**.
Figure S2. HRESIMS data of compound **1**.
Figure S3. ^{1}H NMR spectrum of compound **1** in methanol-d_4.
Figure S4. ^{13}C NMR spectrum of compound **1** in methanol-d_4.
Figure S5. HSQC spectrum of compound **1** in methanol-d_4.
Figure S6. HMBC spectrum of compound **1** in methanol-d_4.
Figure S7. COSY spectrum of compound **1** in methanol-d_4.
Figure S8. IR spectrum of compound **2**.
Figure S9. HRESIMS data of compound **2**.
Figure S10. ^{1}H NMR spectrum of compound **2** in acetone-d_6.
Figure S11. ^{13}C NMR spectrum of compound **2** in acetone-d_6.
Figure S12. HSQC spectrum of compound **2** in acetone-d_6.
Figure S13. HMBC spectrum of compound **2** in acetone-d_6.
Figure S14. IR spectrum of compound **11**.
Figure S15. HRESIMS data of compound **11**.
Figure S16. ^{1}H NMR spectrum of compound **11** in acetone-d_6.
Figure S17. ^{13}C NMR spectrum and DEPT spectrum of compound **11** in acetone-d_6.
Figure S18. HSQC spectrum of compound **11** in acetone-d_6.
Figure S19. HMBC spectrum of compound **11** in acetone-d_6.
Figure S20. COSY spectrum of compound **11** in acetone-d_6.
Figure S21. NOESY spectrum of compound **11** in acetone-d_6.
Figure S22. Effects of compounds **4**, **4b**, **4d**, **6**, and **7** on NO production in LPS-stimulated RAW 264.7 macrophages
Figure S23. Effects of compounds **4**, **4b**, **4d**, **6**, and **7** on cell viability of RAW 264.7 macrophages.

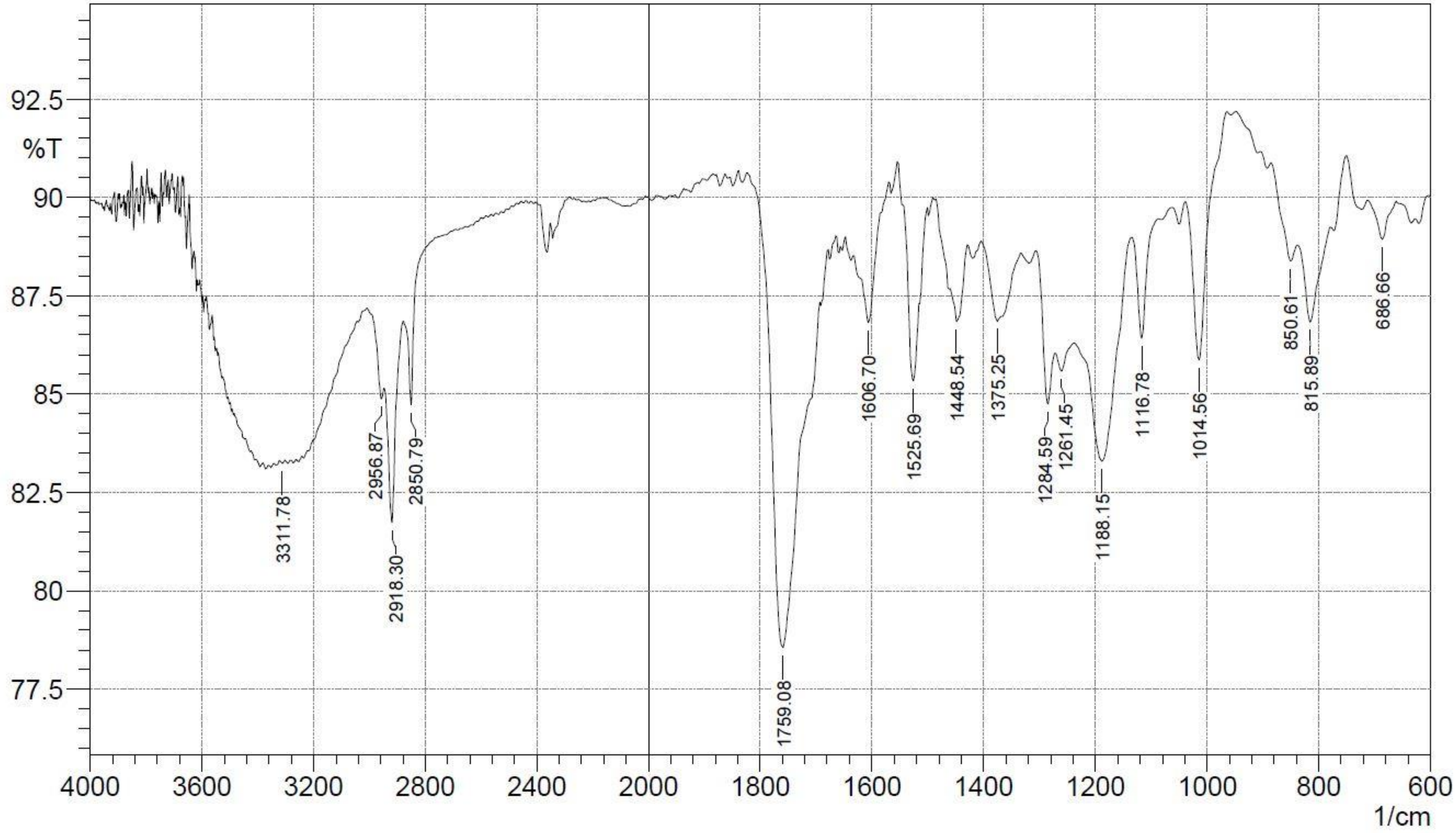

Figure S1. IR spectrum of compound **1**.

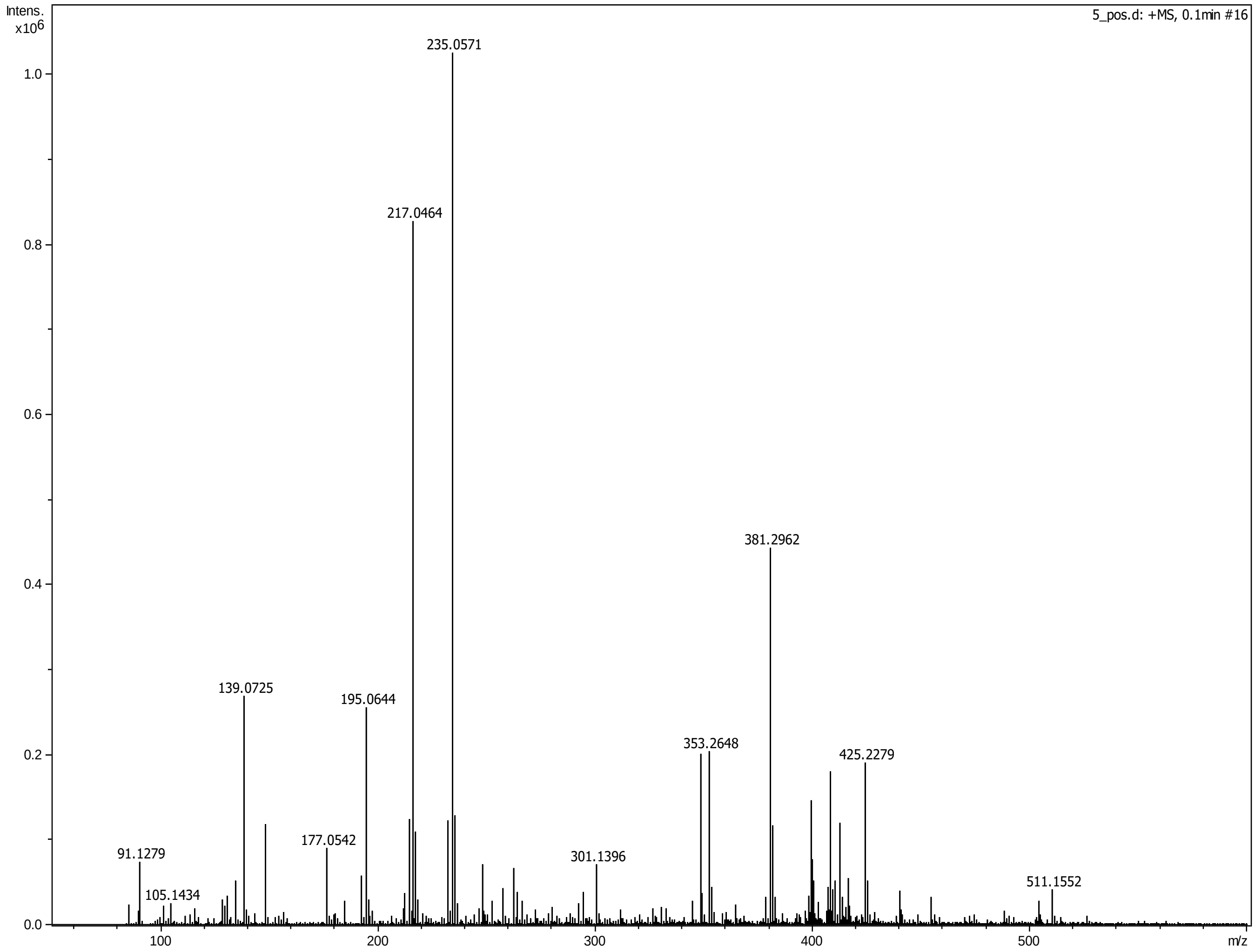

Figure S2. HRESIMS data of compound **1**.

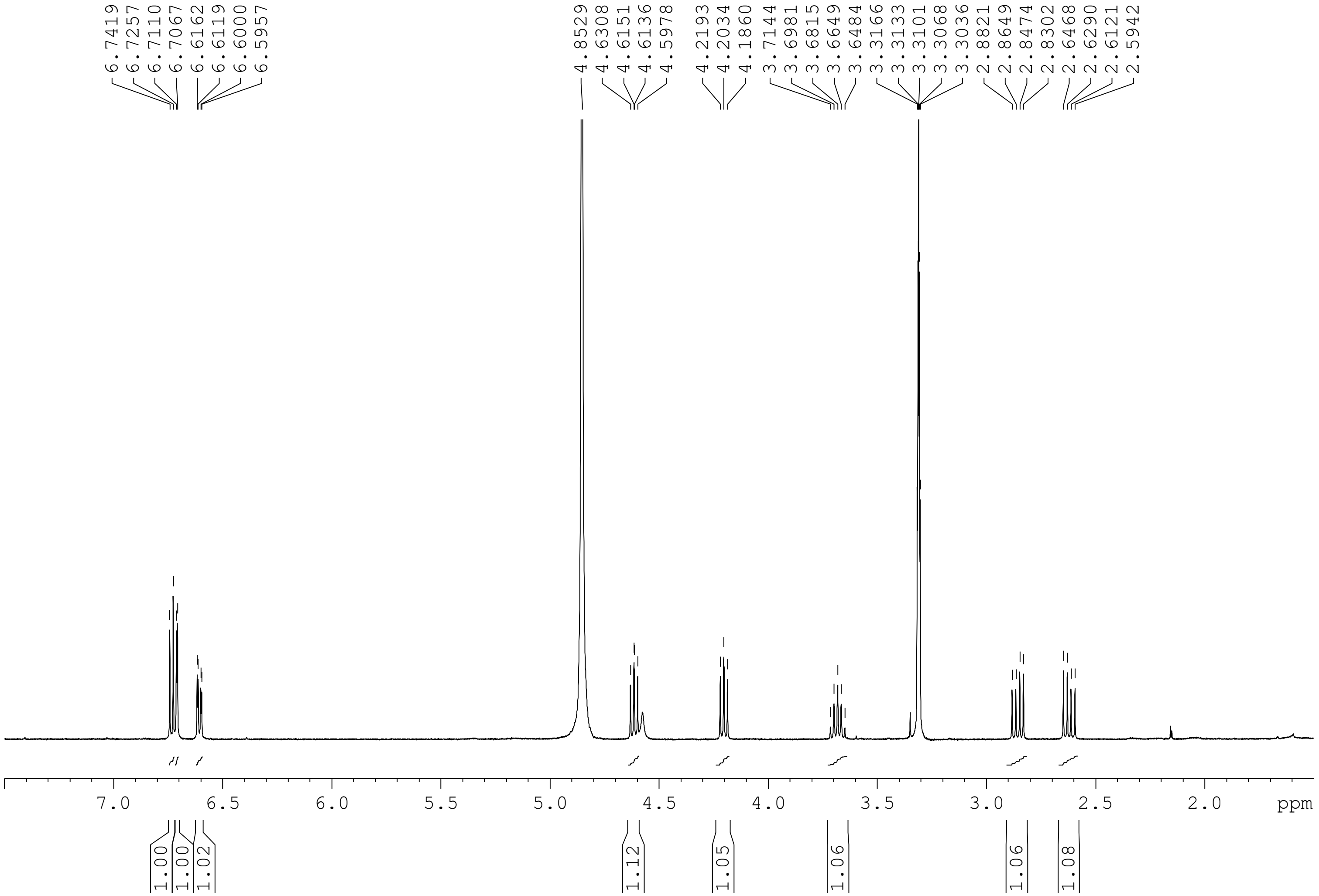

Figure S3. ^{1}H NMR spectrum of compound **1** in methanol-d_4.

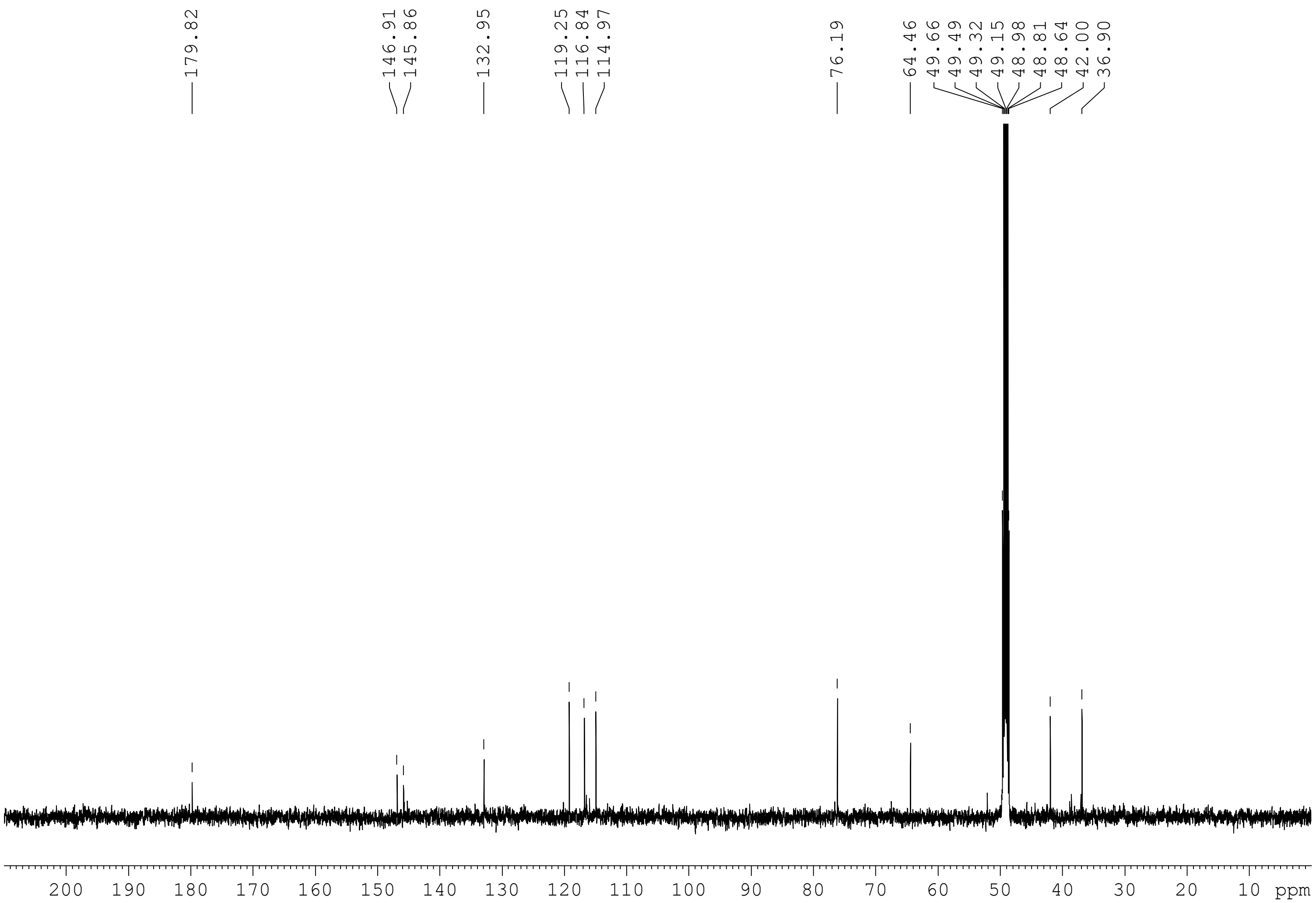

Figure S4. ^{13}C NMR spectrum of compound **1** in methanol-d_4.

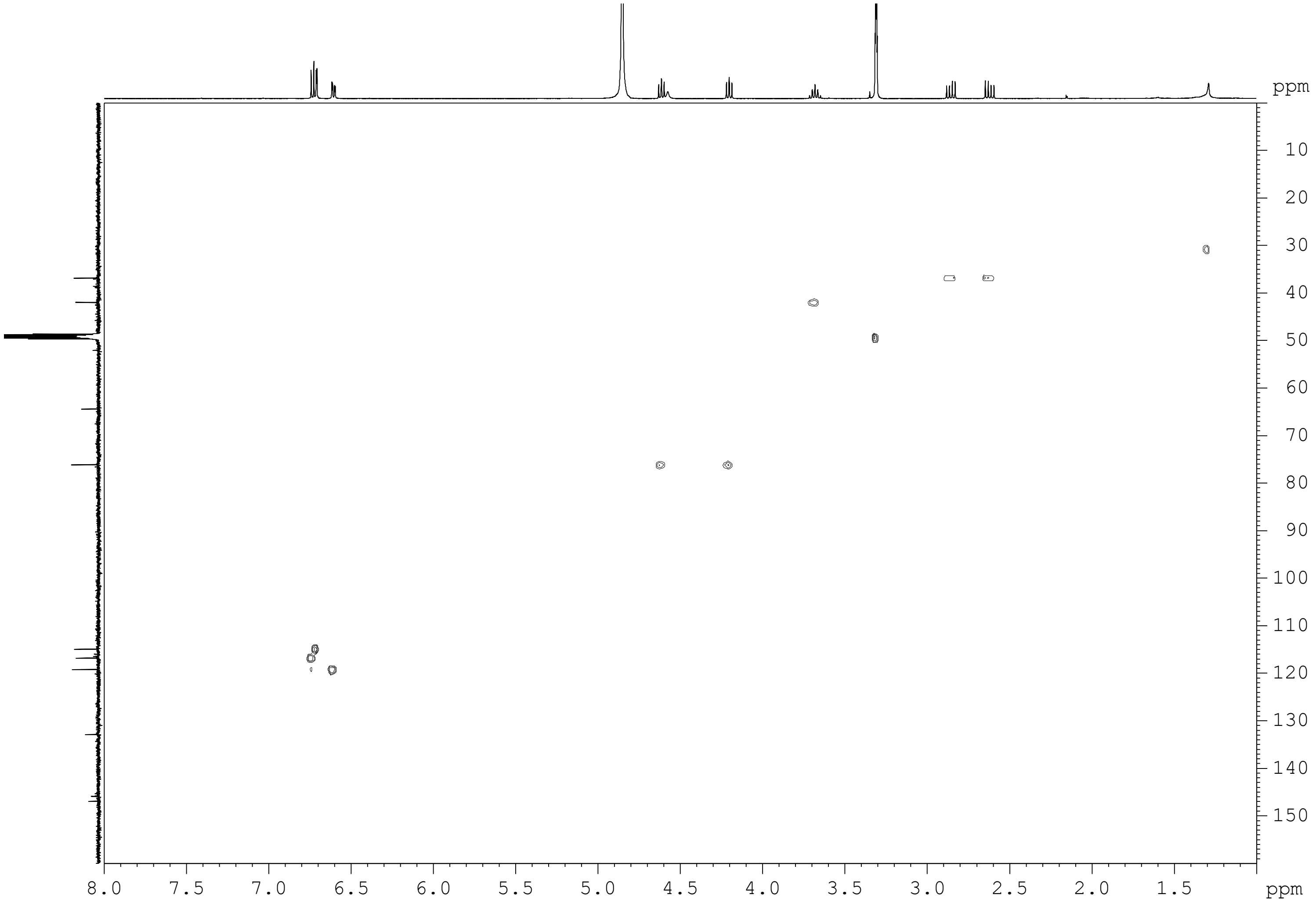

Figure S5. HSQC spectrum of compound **1** in methanol-d_4.

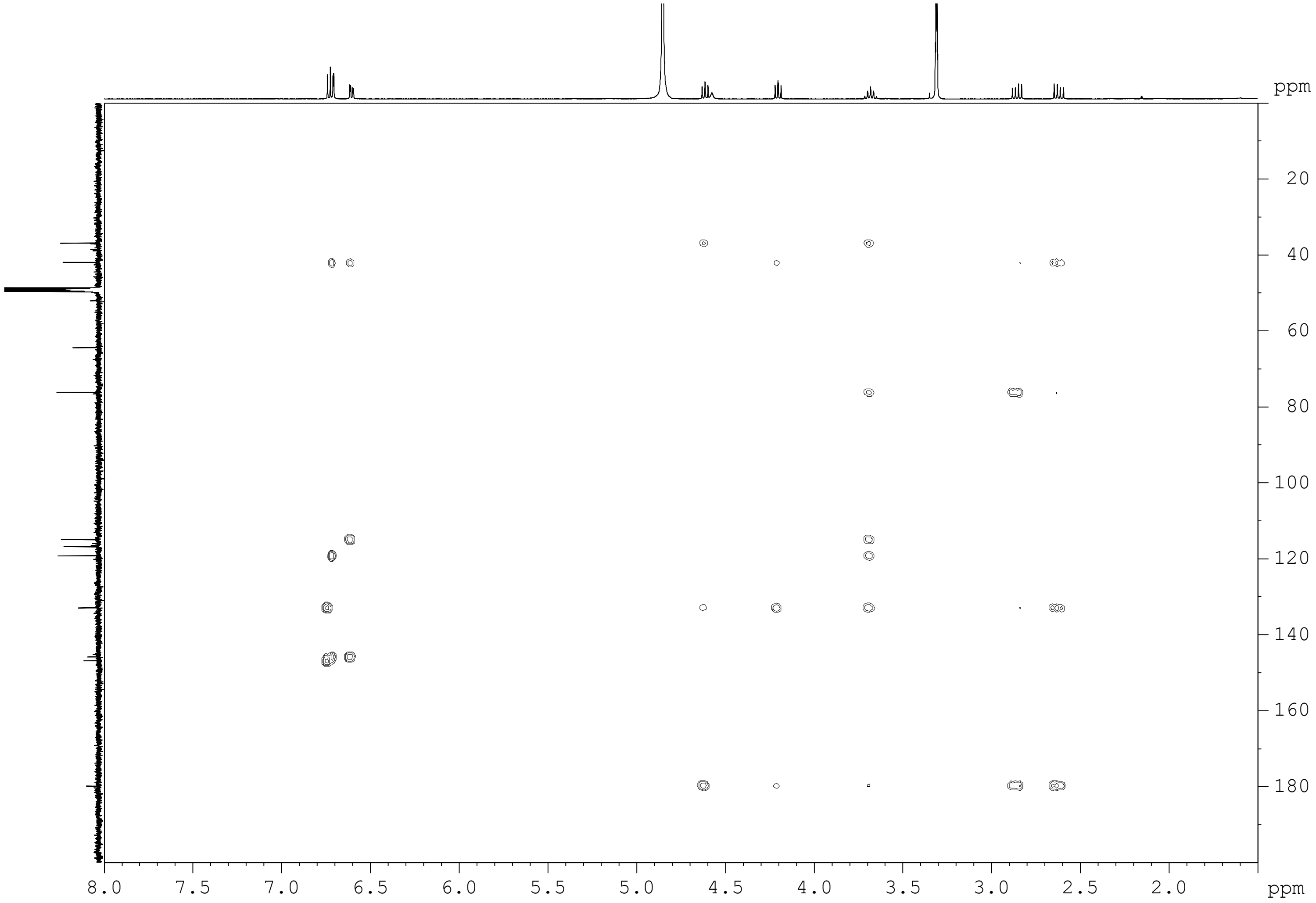

Figure S6. HMBC spectrum of compound **1** in methanol-d_4.

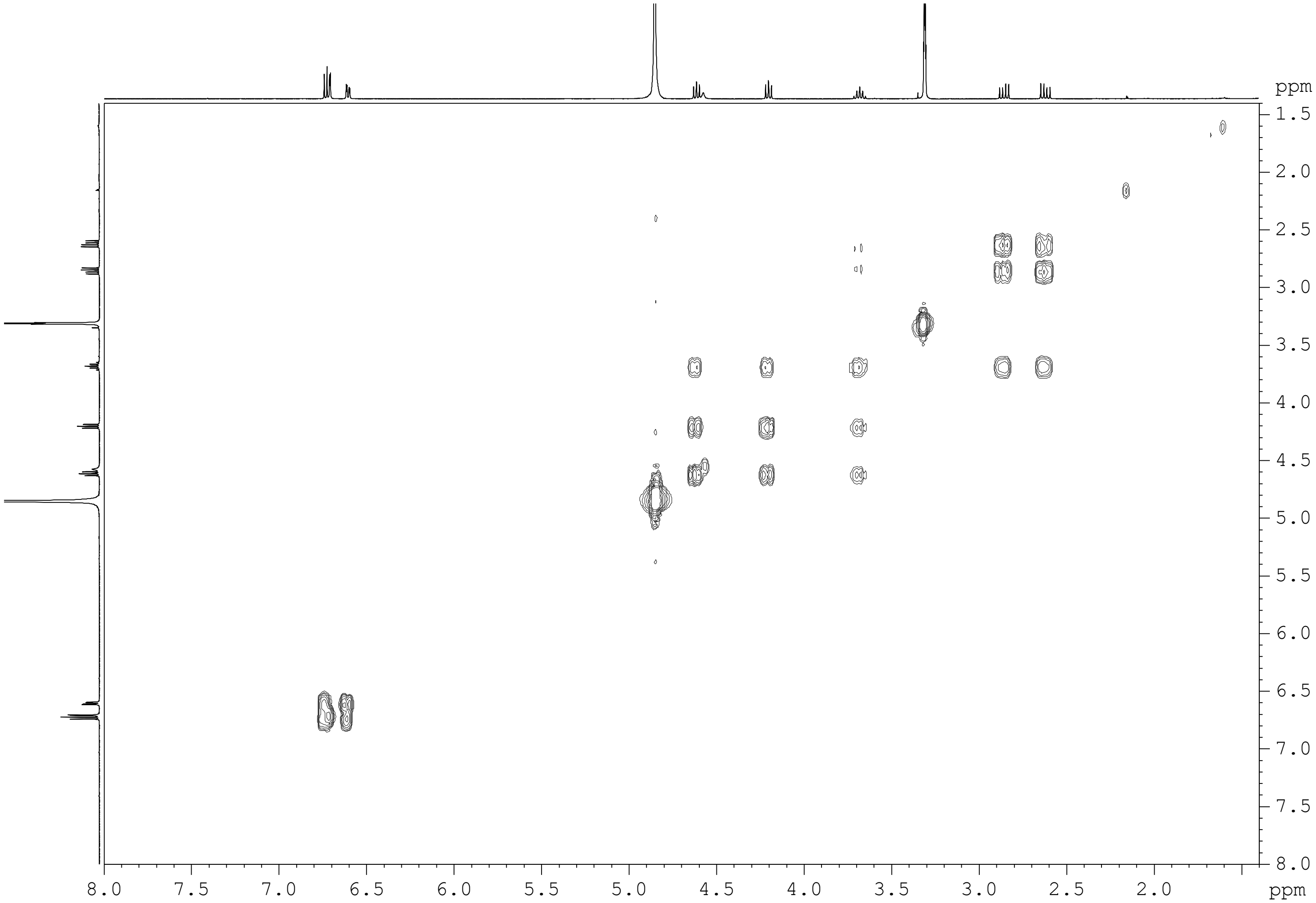

Figure S7. COSY spectrum of compound **1** in methanol-d_4.

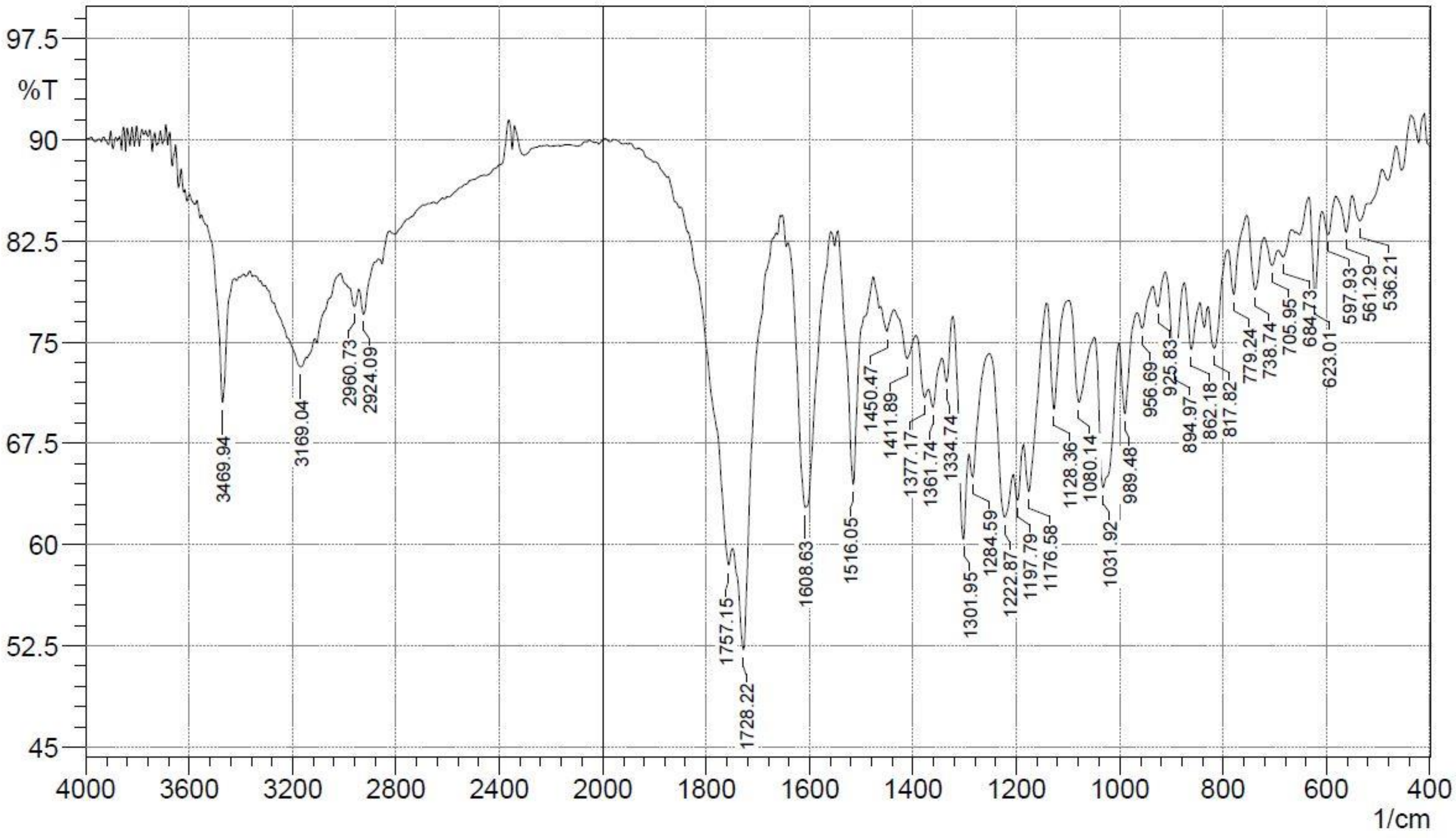

Figure S8. IR spectrum of compound **2**.

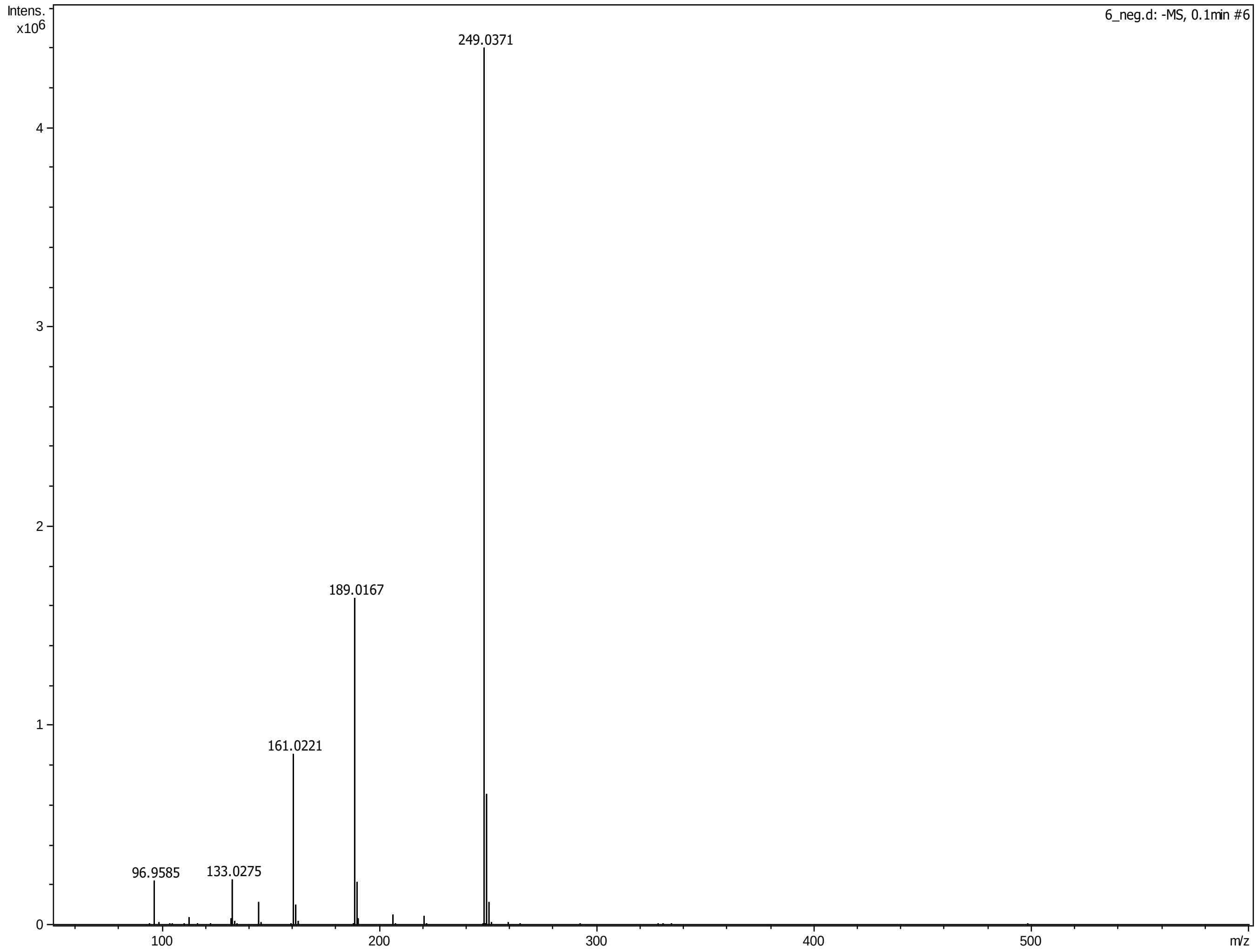

Figure S9. HRESIMS data of compound **2**.

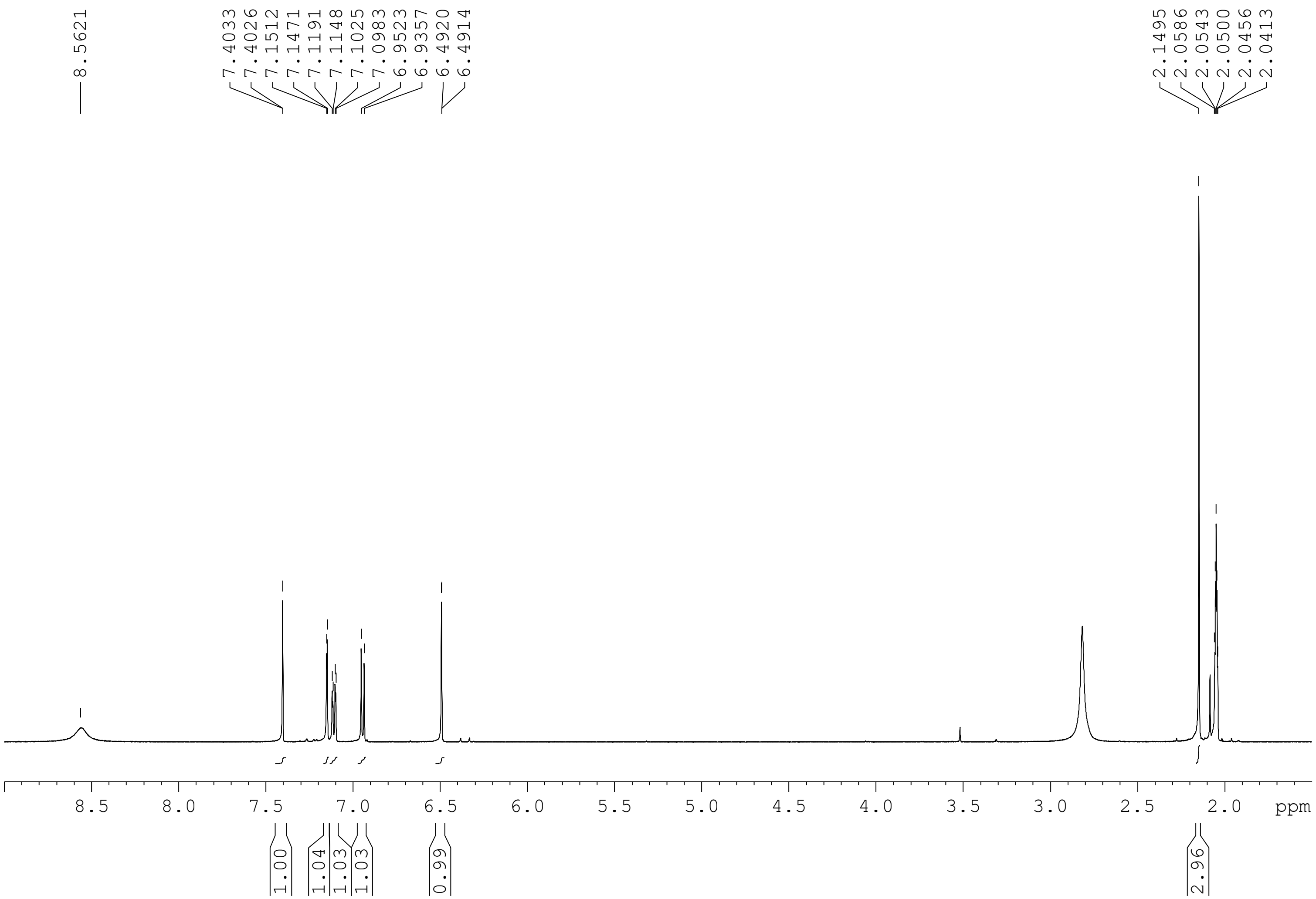

Figure S10. ^{1}H NMR spectrum of compound **2** in acetone-d_6.

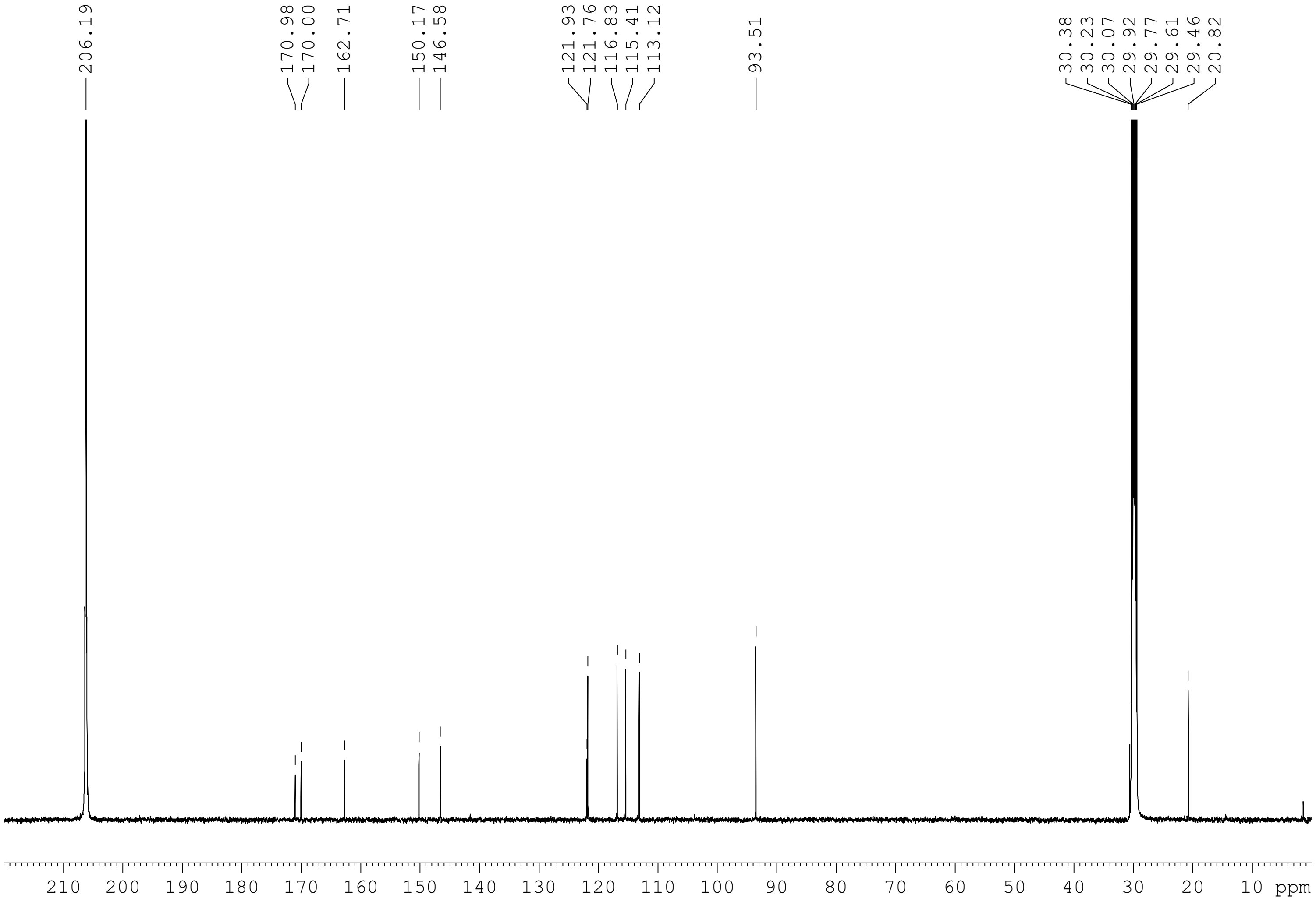

Figure S11. ^{13}C NMR spectrum of compound **2** in acetone-d_6.

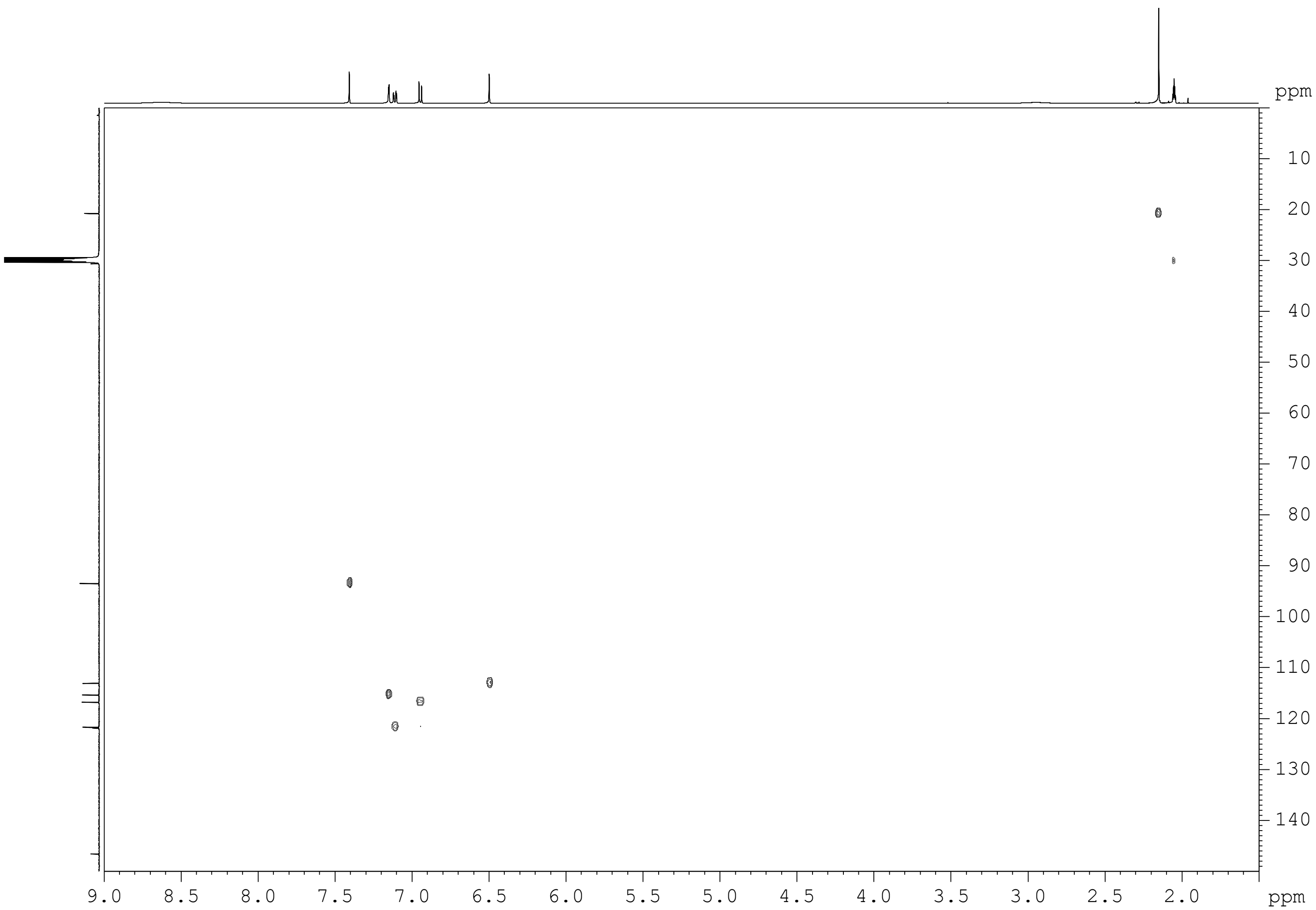

Figure S12. HSQC spectrum of compound **2** in acetone-d_6.

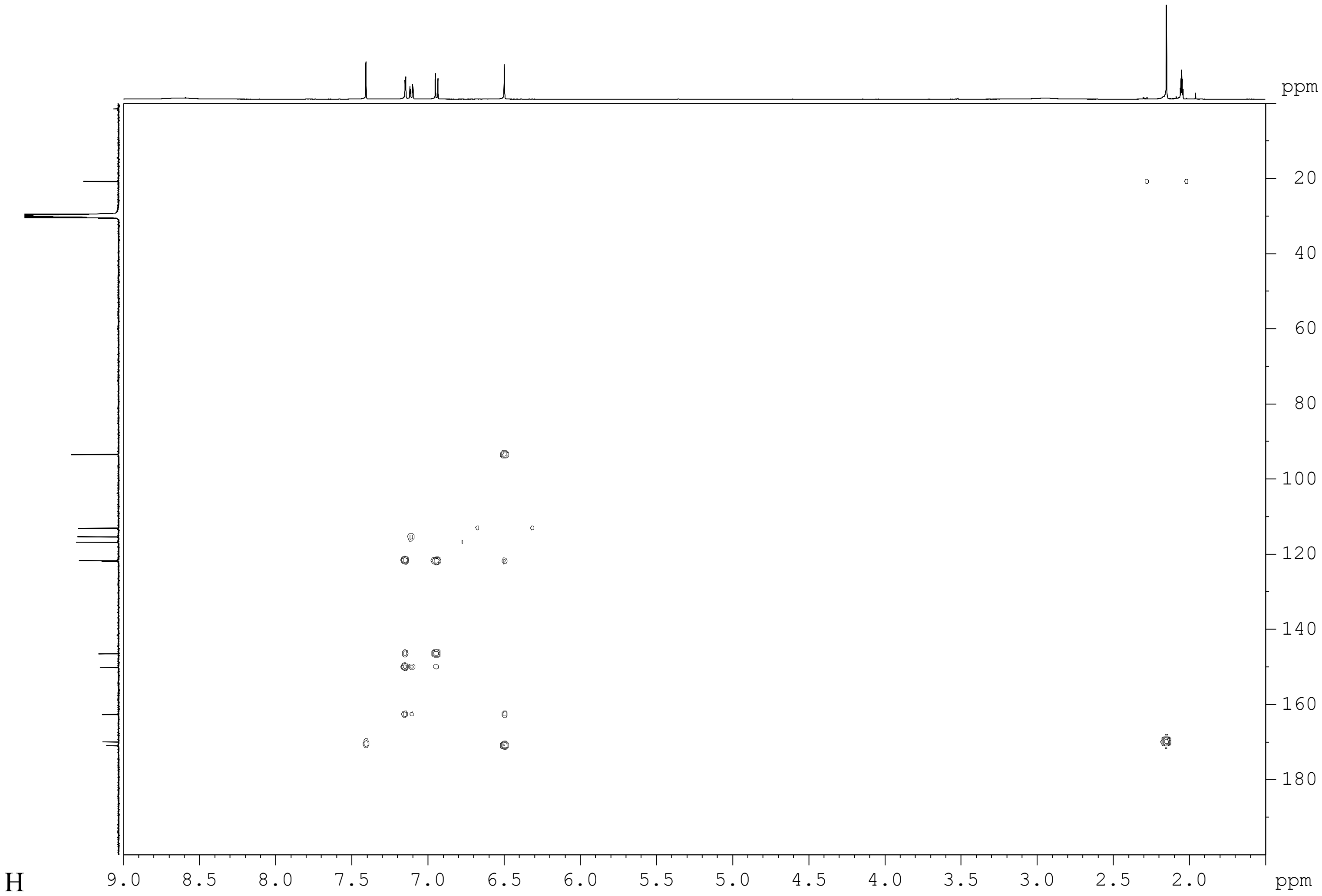

Figure S13. HMBC spectrum of compound **2** in acetone-d_6.

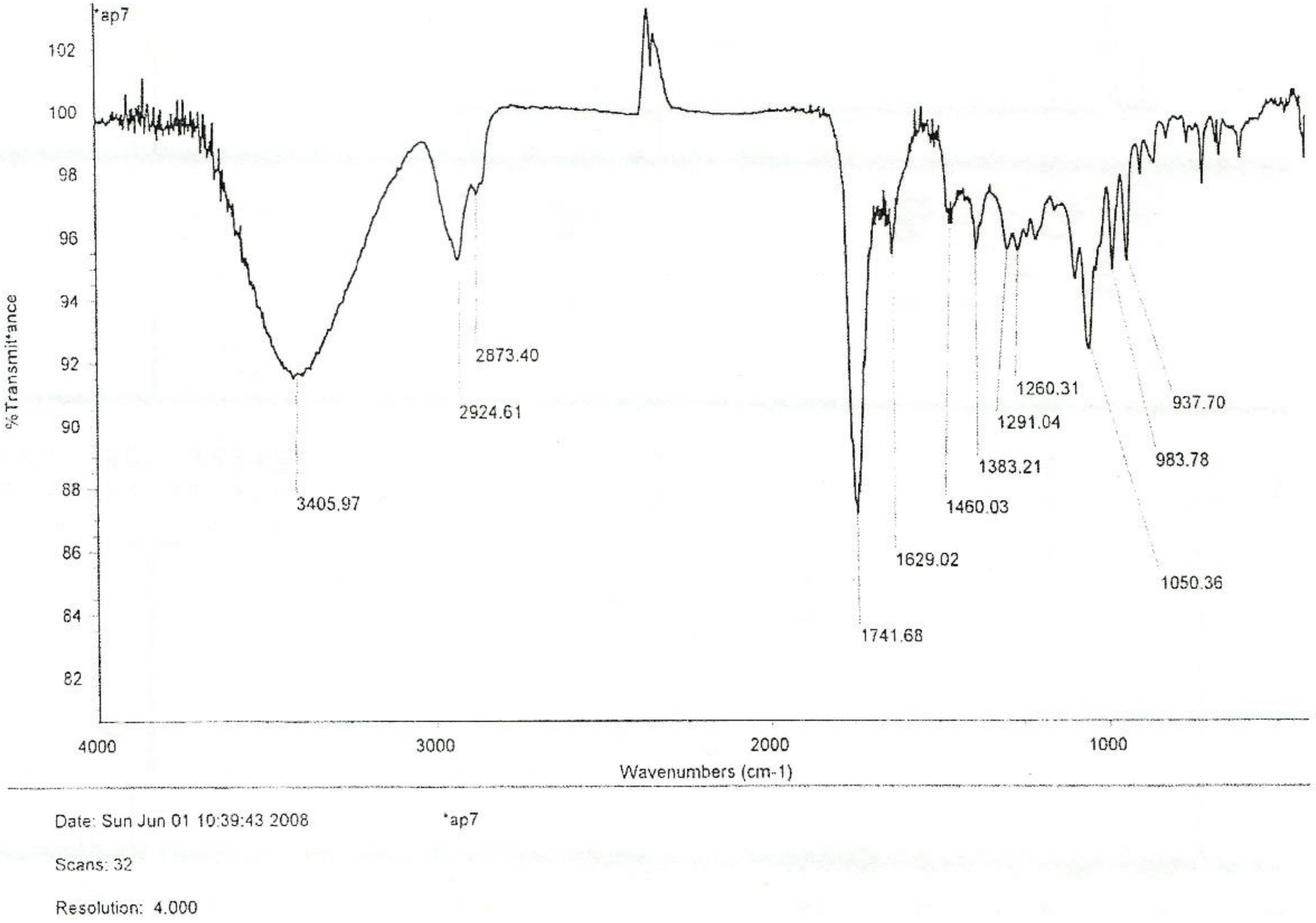

Figure S14. IR spectrum of compound **11**.

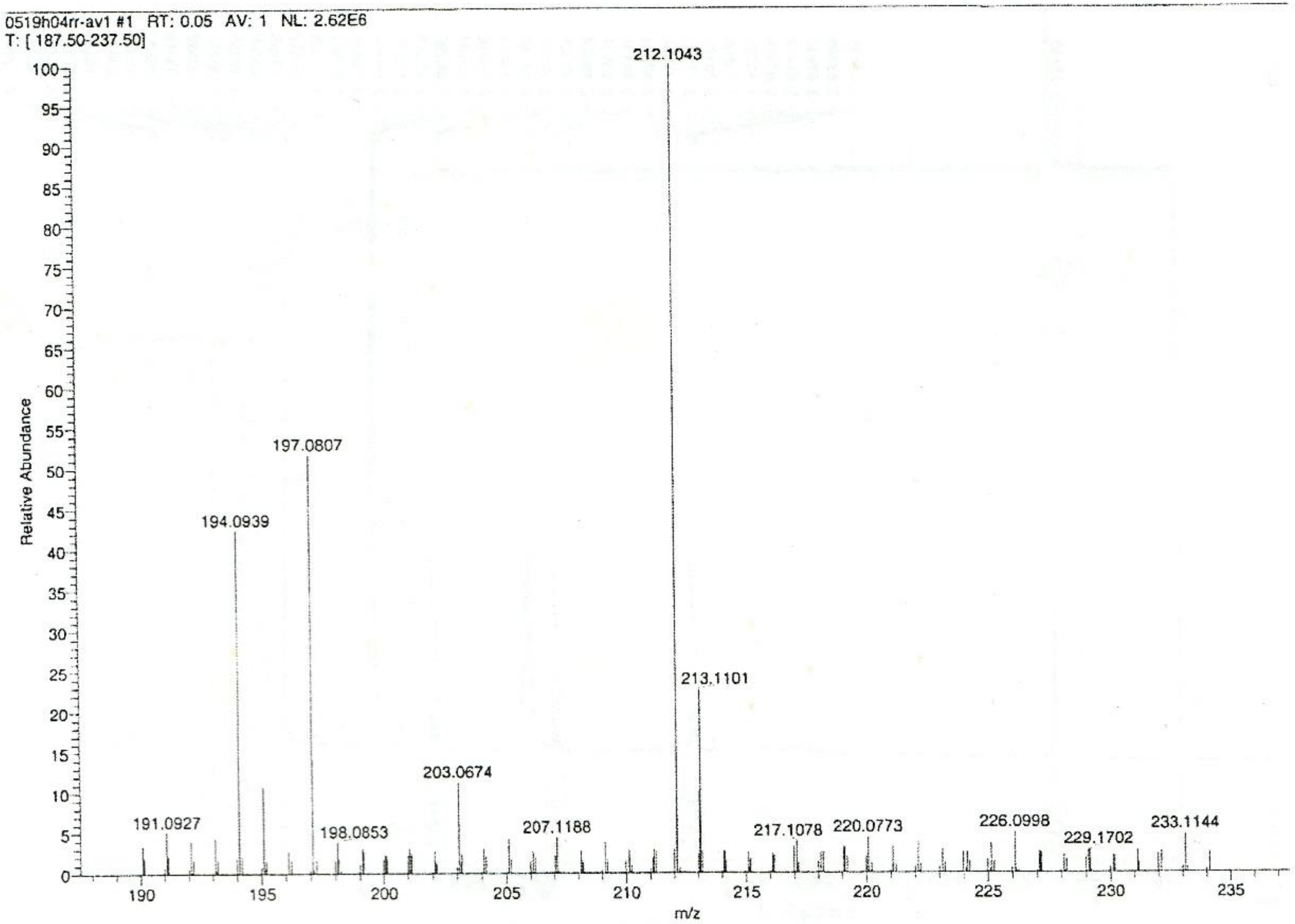

Figure S15. HRESIMS data of compound **11**.

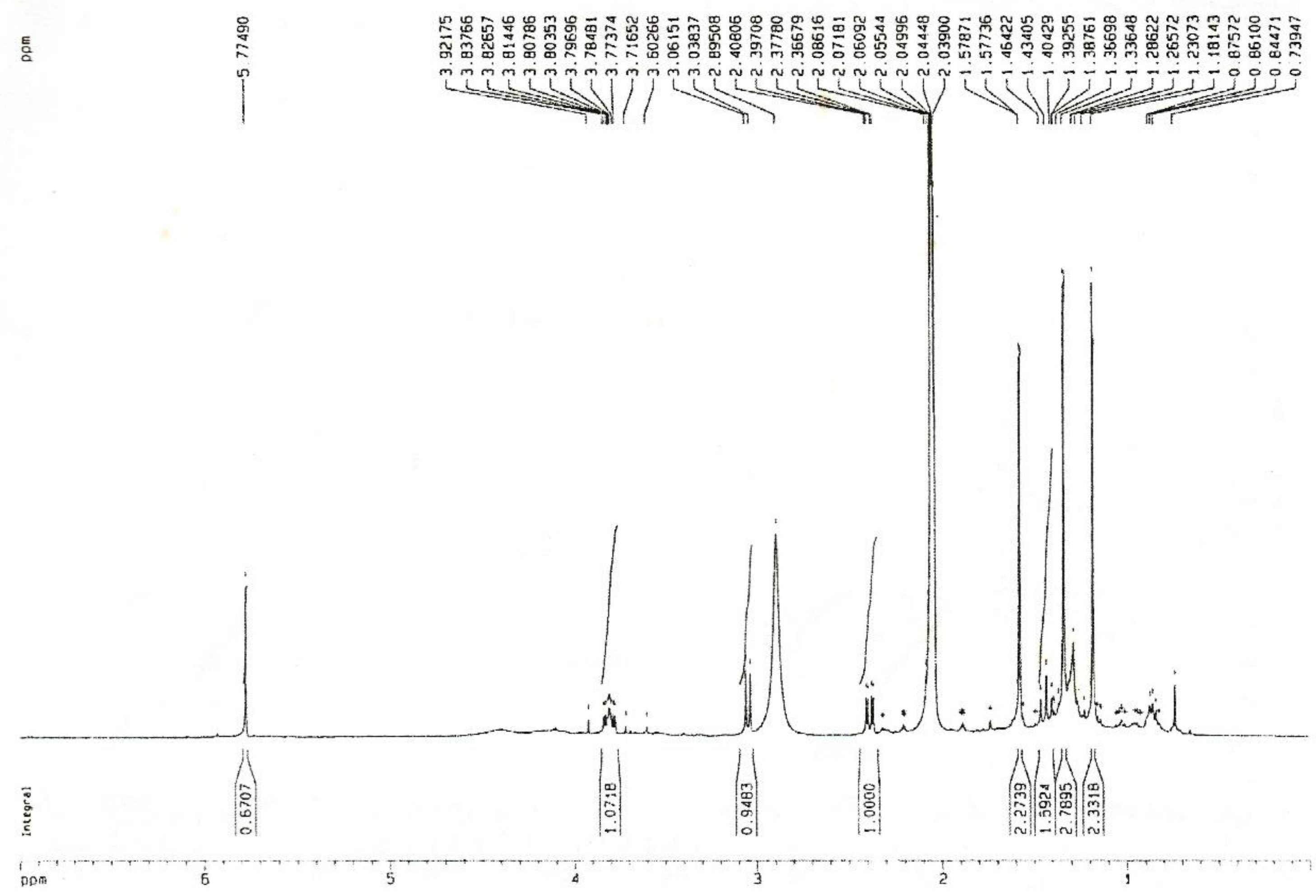

Figure S16. ^{1}H NMR spectrum of compound **11** in acetone-d_6.

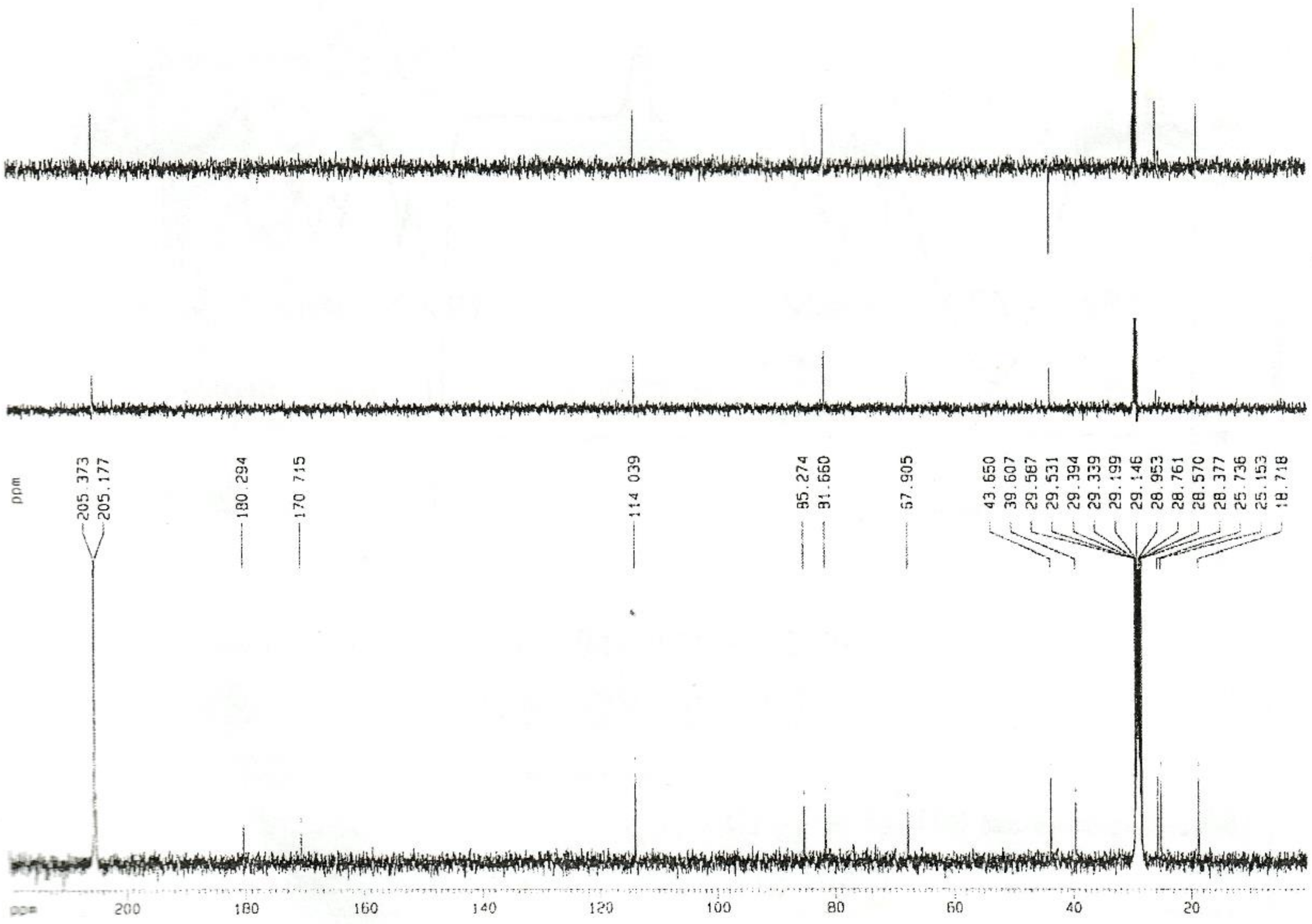

Figure S17. ^{13}C NMR spectrum and DEPT spectrum of compound **11** in acetone-d_6.

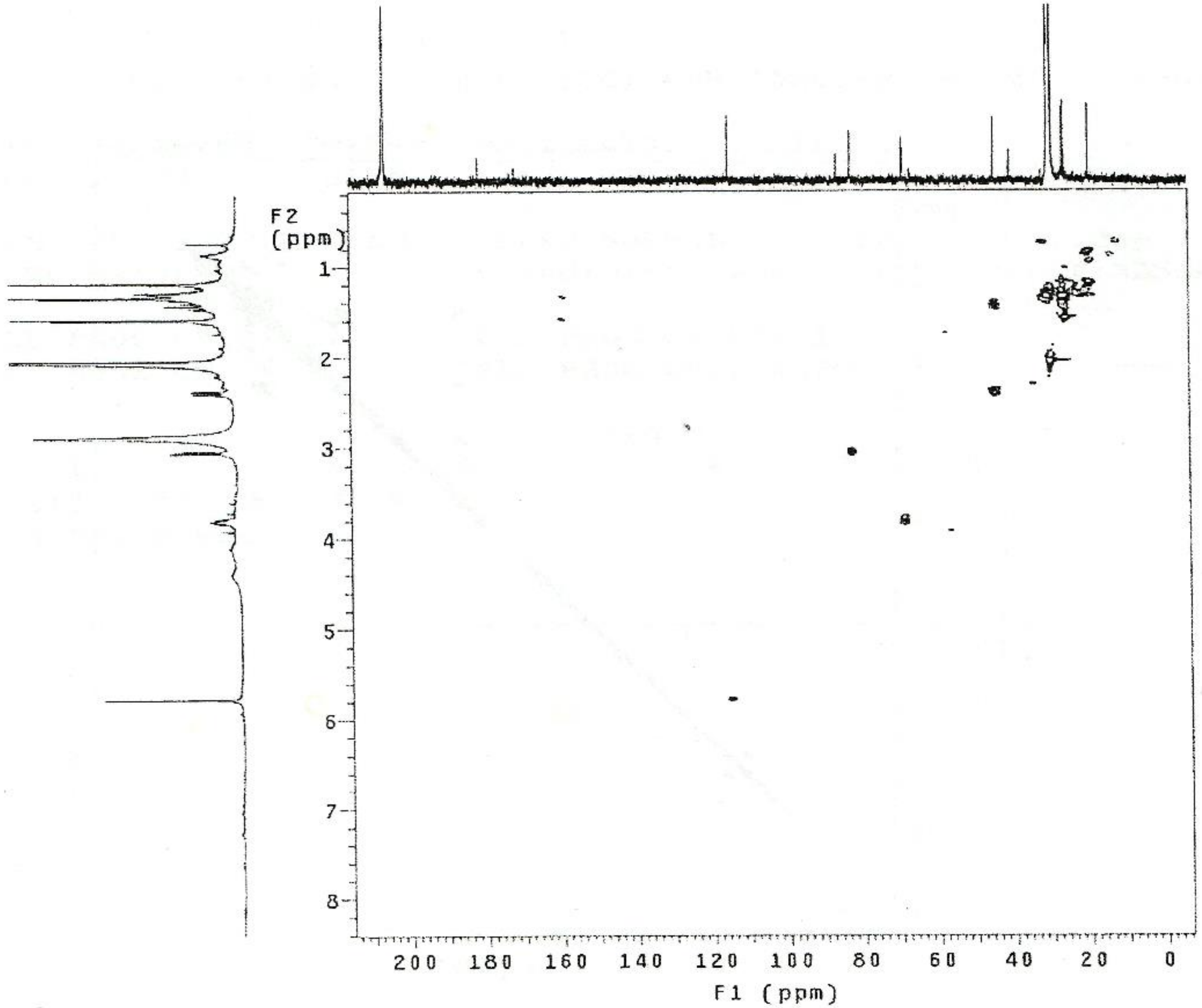

Figure S18. HSQC spectrum of compound **11** in acetone-d_6.

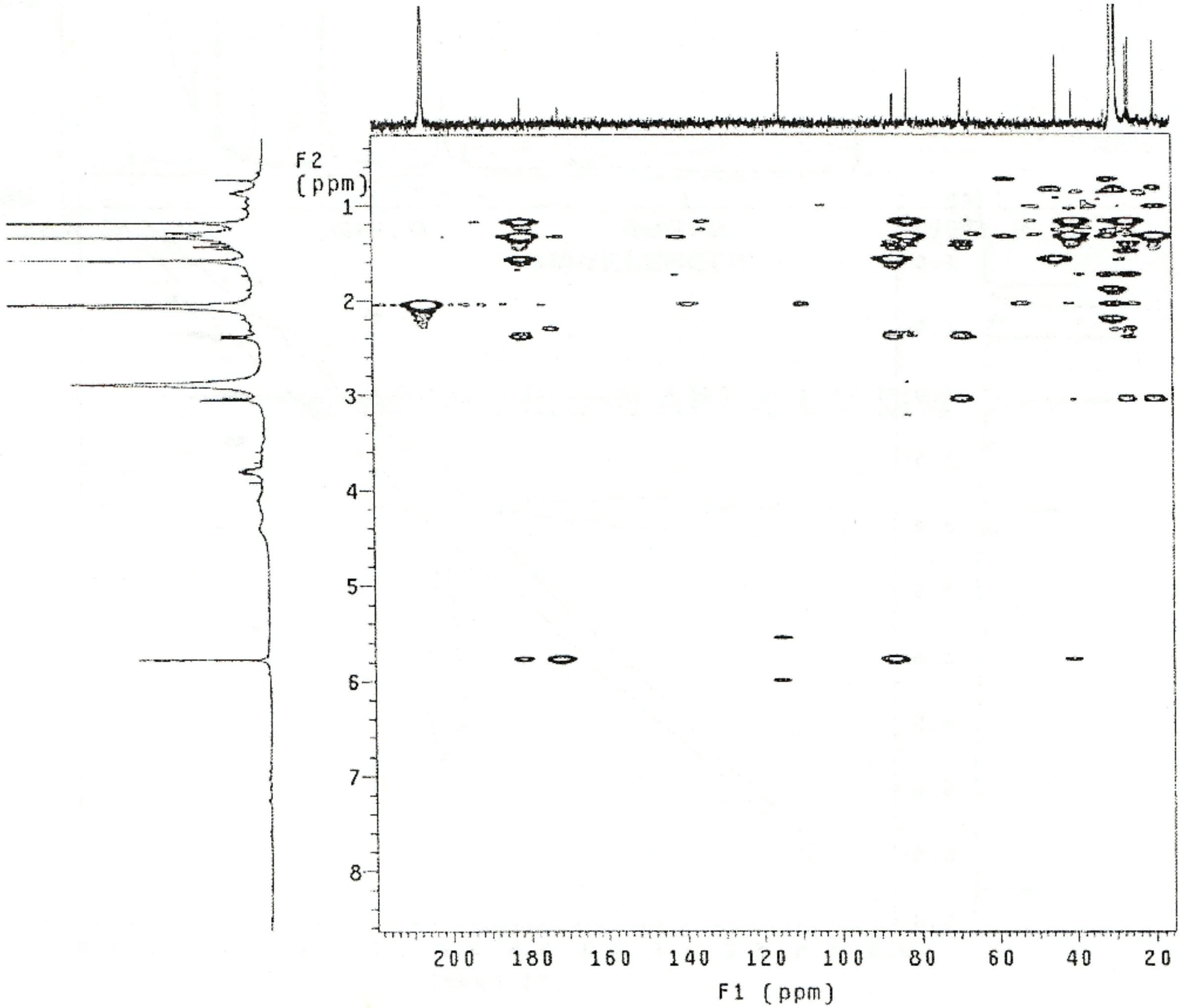

Figure S19. HMBC spectrum of compound **11** in acetone-d_6.

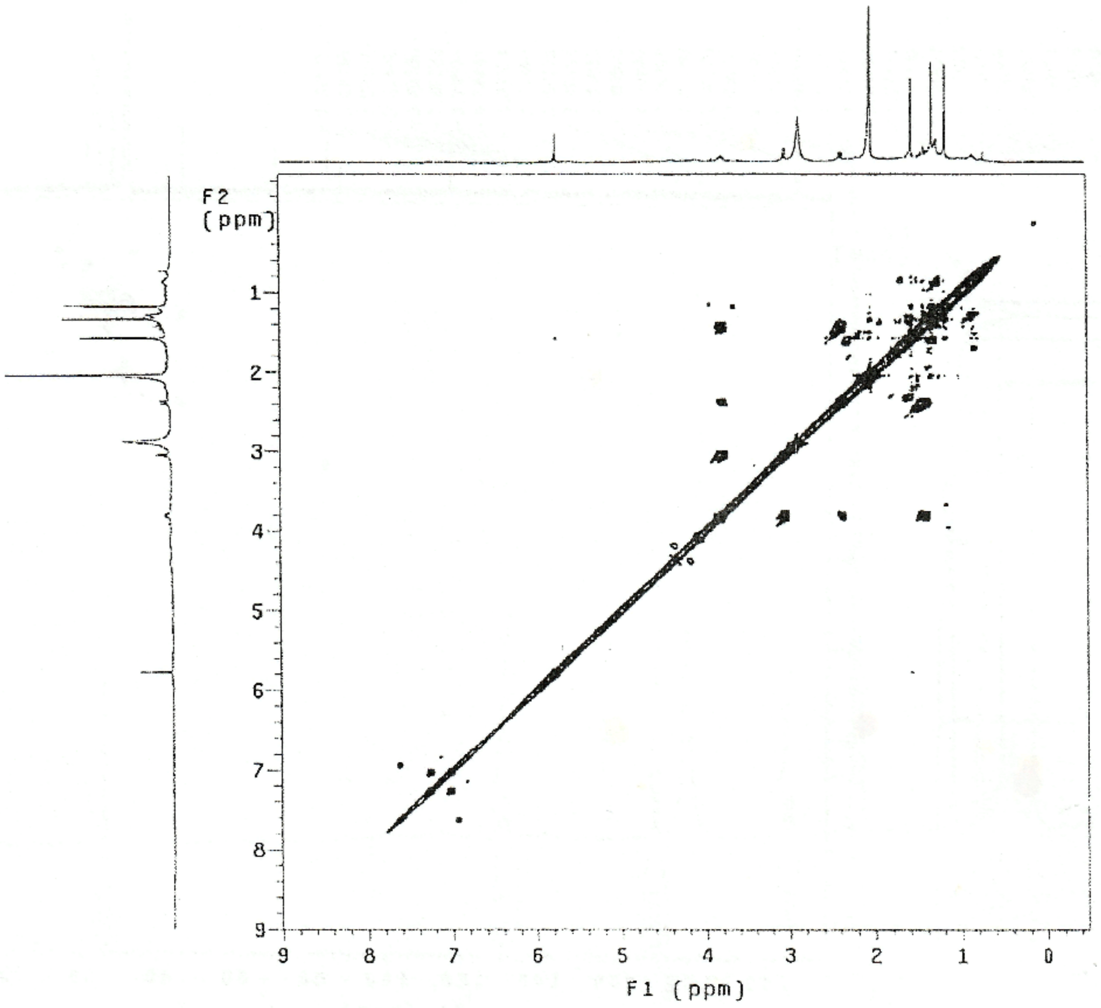

Figure S20. COSY spectrum of compound **11** in acetone-d_6.

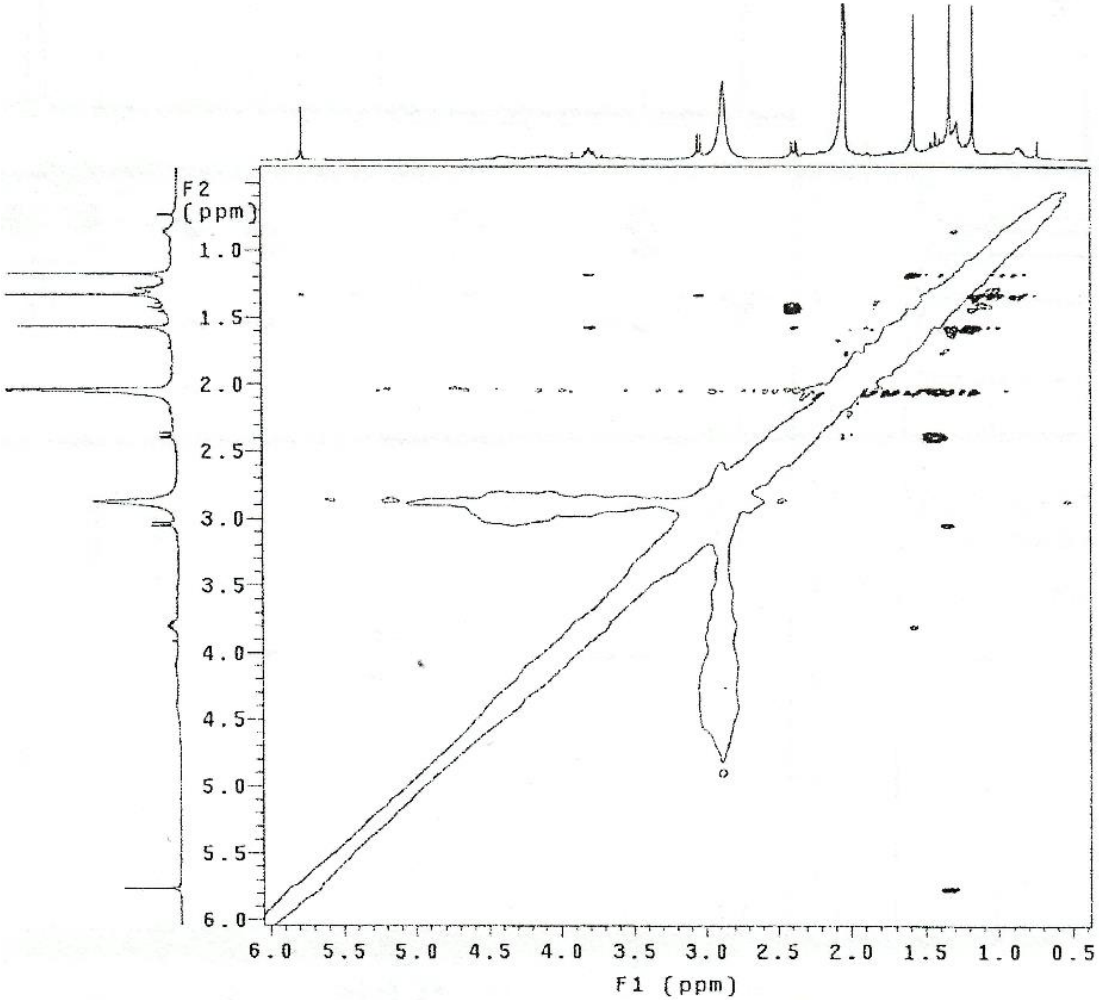

Figure S21. NOESY spectrum of compound **11** in acetone-d_6.

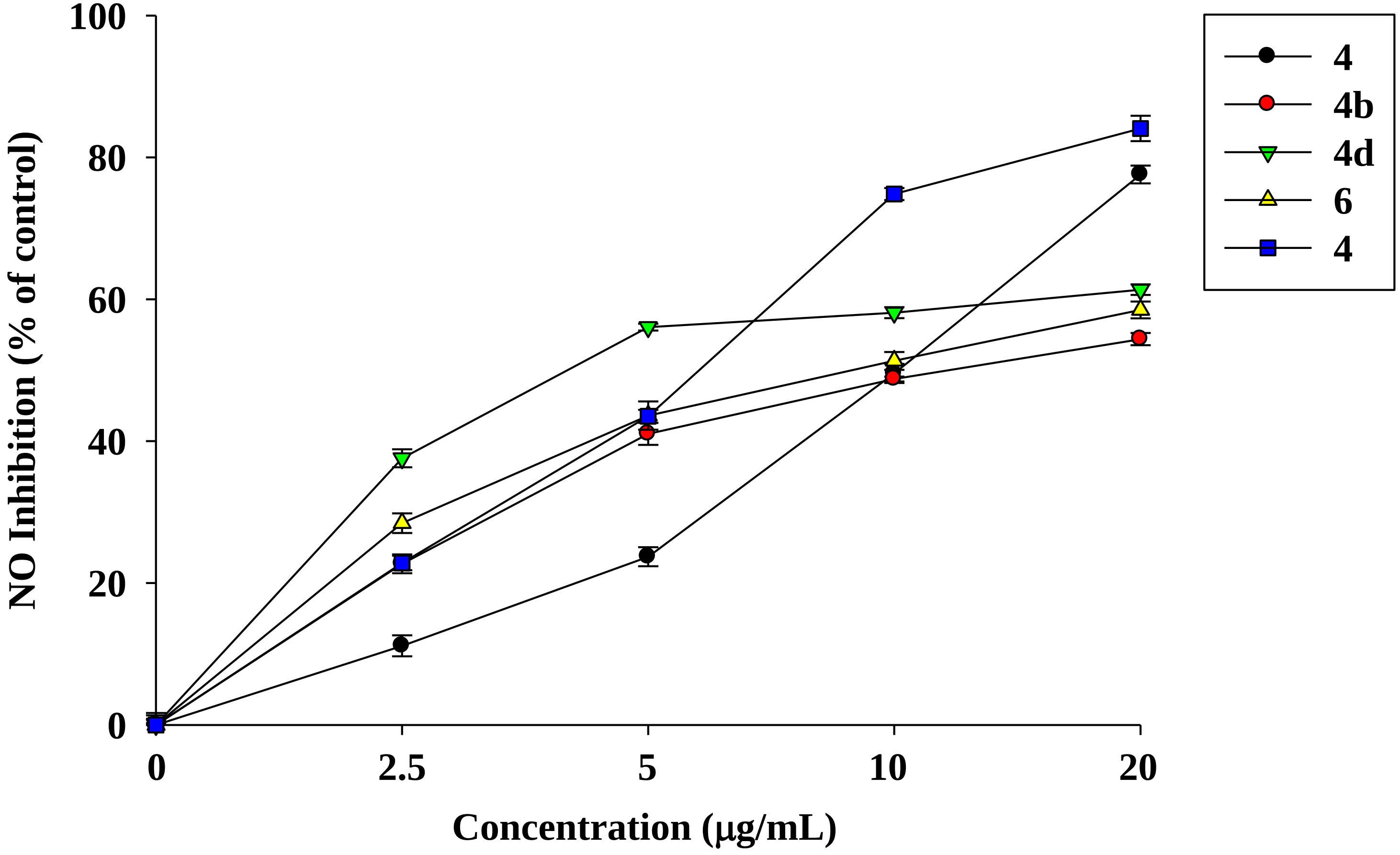

Figure S22. Effects of compounds **4**, **4b**, **4d**, **6**, and **7** on NO production in LPS-stimulated RAW 264.7 macrophages

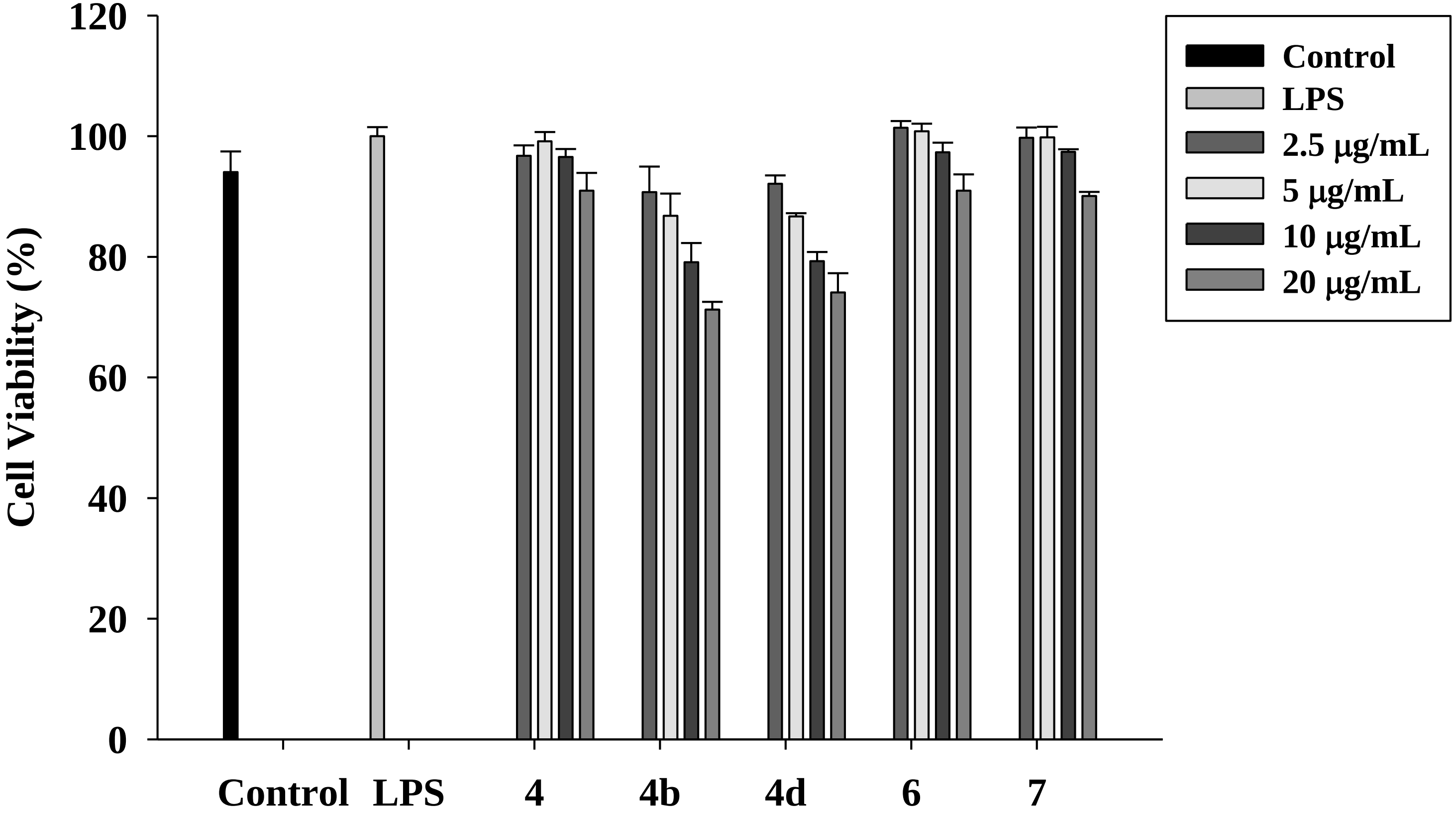

Figure S23. Effects of compounds **4**, **4b**, **4d**, **6**, and **7** on cell viability of RAW 264.7 macrophages.
